# Acute pulmonary embolism in the era of multi-detector CT: a reality in sub-Saharan Africa

**DOI:** 10.1186/1471-2342-12-31

**Published:** 2012-10-17

**Authors:** Joshua Tambe, Boniface Moifo, Emmanuel Fongang, Emilienne Guegang, Alain Georges Juimo

**Affiliations:** 1 Department of Radiology and Radiation Oncology, University of Yaounde 1, Yaounde, Cameroon; 2 Yaounde General Hospital, Yaounde, Cameroon

**Keywords:** Acute pulmonary embolism, multi-detector CT angiography, sub-Saharan Africa, thromboembolic risk factors

## Abstract

**Background:**

The advantages of multi-detector computed tomography (MDCT) have made it the imaging modality of choice for some patients with suspected cardiothoracic disease, of which pulmonary embolism (PE) is an exponent. The aim of this study was to assess the incidence of PE in patients with clinical suspicion of acute PE using MDCT in a sub-Saharan setting, and to describe the demographic characteristics of these patients.

**Methods:**

Consecutive records of patients who underwent MDCT pulmonary angiography for suspected acute PE over a two-year period at the Radiology Department of a university-affiliated hospital were systematically reviewed. All MDCT pulmonary angiograms were performed with a 16-detector computed tomography (CT) scanner using real-time bolus tracking technique. Authorization for the study was obtained from the institutional authorities.

**Results:**

Forty-one MDCT pulmonary angiograms were reviewed of which 37 were retained. Of the 4 excluded studies, 3 were repeat angiograms and 1 study was not technically adequate. Twelve of 37 patients (32.4%) had CT angiograms that were positive for PE, of which 7 were males. The mean age of these patients was 47.6±10.5 years (age range from 33 to 65 years). Twenty five patients out of 37 (67.6%) had CT angiograms that were negative for PE. Eleven PE-positive patients (91.7%) had at least 1 identifiable thromboembolic risk factor whilst 5 PE-negative patients (20%) also had at least a thromboembolic risk factor. The relative risk of the occurrence of PE in patients with at least a thromboembolic risk factor was estimated at 14.4.

**Conclusion:**

Acute PE is a reality in sub-Saharan Africa, with an increased likelihood of MDCT evidence in patients with clinical suspicion of PE who have at least a thromboembolic risk factor. The increasing availability of MDCT will help provide more information on the occurrence of PE in these settings.

## Background

Multi-detector computed tomography (MDCT) pulmonary angiography is increasingly being used to evaluate patients with suspected pulmonary embolism (PE) [1]. This is largely due to its many advantages over conventional angiography whose place as a diagnostic standard of reference has been increasingly challenged [2]. MDCT angiography is non-invasive, results in shorter acquisition times with improved contrast enhancement, thinner collimation hence superior image resolution and improved visualization of the pulmonary arterial tree [3-5]. Some studies have shown MDCT pulmonary angiography to have a high diagnostic yield and to be more cost-effective than conventional angiography in investigating patients with suspected PE [6,7]. The diagnostic usefulness of MDCT pulmonary angiography is further enhanced by its ability to detect concurrent or other cardiothoracic diseases mimicking PE. These combined advantages of MDCT angiography make it very appropriate for some patients with suspected cardiothoracic disease [1,8].

Little is known about the occurrence of acute pulmonary embolism (PE) in sub-Saharan settings. Considered rare, it has often been diagnosed based on clinical symptoms in patients with or without evidence of deep vein thrombosis on Doppler ultrasound [9]. This has largely been due to the lack of diagnostic facilities over the years. With the introduction of MDCT, it has proved to be a useful diagnostic tool in investigating suspected cases of acute PE. The purpose of this study therefore was to assess the incidence of PE in patients with clinical suspicion of acute PE using MDCT in a sub-Saharan setting, and to describe the demographic characteristics of these patients.

## Results

A total of 41 CT pulmonary angiograms were reviewed, of which 37 were retained. Of the 4 excluded studies, 3 were repeat CT angiograms, and in 1 study the pulmonary arteries were not sufficiently opacified.

### Patient demographics

The mean age of the study population was 48.5±12.7 years (mean±SD) with range from 23 to 74 years, and sex-ratio 1:1. Twelve of 37 patients (32.4%) had CT angiograms that were positive for PE, with a mean age of 47.6±10.5 years (range 33 to 65 years). Table 1 summarizes the demographic characteristics of the study population.

**Table 1:** Demographic characteristics of the study population

	**All Patients**	**PE positive**	**PE negative**
Age (years)*			
**All**	48.5±12.7	47.6±10.6	48.9±13.8
**Male**	52.5±11.2	46.1±10.1	57.0±13.5
**Female**	45.1±13.1	49.6±12.0	43.6±10.1
**Age range**	23 – 74	33 – 65	23 – 74
**Sex, no./total no.(%)**			
**Male**	17/37 (45.9)	7/12 (58.3)	10/25 (40.0)
**Female**	20/37 (54.1)	5/12 (41.7)	15/25 (60.0)

* Plus-minus values are mean±standard deviation (SD).

### Risk factors

The specific thromboembolic risk factors identified in the study population with their associated frequencies are shown in Table 2.

**Table 2:** Identified thromboembolic risk factors and frequencies

**Risk factor**	**PE-positive**	**PE-negative**
**Age ≥ 65 years**	1	3
**Postoperative state**	5	2
**Known DVT†**	3	0
**Malignancy**	1	0
**Immobilisation for medical reasons**	4	2

DVT† stands for deep vein thrombosis.

**Figure 1:**
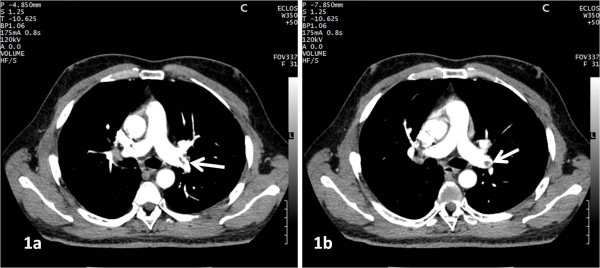
**A 41-year-old man, with dyspnea of sudden onset. **CT angiogram shows a typical embolus in the left pulmonary artery (arrow). No thromboembolic risk factor was found.

Of the 12 patients with CT angiograms positive for PE, 11 (91.7%) had at least one identifiable thromboembolic risk factor. One patient had no identifiable thromboembolic risk factor. Five patients with PE-negative scans (20%) had at least 1 identifiable thromboembolic risk factor. The relative risk of the occurrence of PE with respect to the thromboembolic risk factors was 14.4 *(RR =11/16 × 21 = 14.4)* (Figure 1).

### Ancillary and other significant CT angiographic findings of PE-positive and PE-negative patients

**Figure 2:**
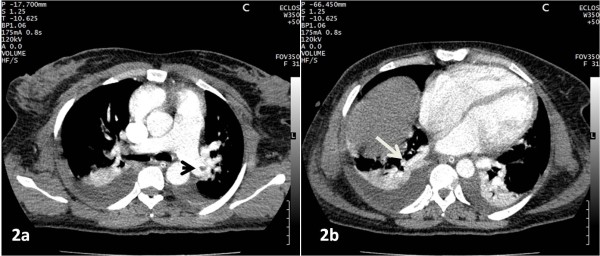
**CT angiogram of a 33-year-old woman with clinical suspicion of PE three days after surgery (Caesarean section) shows an embolus in the left pulmonary artery (2a, arrowhead) and in the right postero-basal pulmonary artery (2b, white arrow). **Note bilateral pleural effusion and peripheral wedge-shaped opacity.

**Figure 3:**
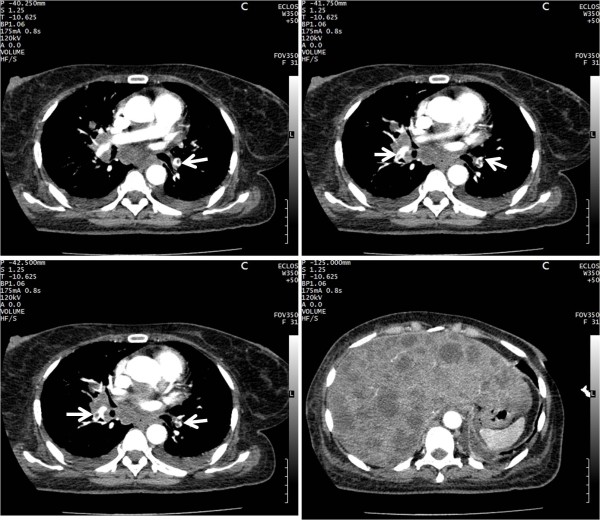
**CT angiogram in a 50-year-old woman with sudden and unexplained shortness of breath, who was also restless, shows multiple and bilateral emboli (3a, 3b, 3c, white arrow) associated with multiple enlarged mediastinal and hilar lymph nodes. **The liver is enlarged with multiple hypodense and non-enhancing nodules suggestive of metastases (3d).

Ancillary findings were observed in some PE-positive patients: they included 4 cases of wedge-shaped opacities with pleural effusions and 2 cases of pleural effusions only. Significant findings in PE-negative patients included 5 cases of lung consolidation, 2 cases of ground glass opacities, 4 cases of pleural effusions, a case of pericardial effusion, 3 cases of aneurysms of the thoracic aorta and a case of aneurysm of the main pulmonary artery (Figures 2 and 3).

## Discussion

From these results acute PE is not as rare a finding in sub-Saharan African populations as previously reported [9], after obtaining as high as 32% MDCT angiograms that were positive for PE. This positivity rate exceeds that of the multicentre PIOPED II (prospective investigation of pulmonary embolism diagnosis) study which recorded 23.3% positive scans for PE [10,11]. Mamlouk et al. and Prologo et al. working in the United States recorded even lower rates of 9.8% and 10.4% respectively [12,13] whilst Karabulut and Kiroglu in Turkey obtained 38% [14]. This study was carried out in just one hospital setting which alone had the available diagnostic imaging facility (16-detector CT) in the locality. This relative unavailability of diagnostic imaging facilities imposes a geographic selection as eligible patients with clinical suspicion of PE in settings without this facility would not have had access to MDCT. Also, some eligible patients in the setting where this facility is available might not have had access due to the cost (the direct cost of a CT angiogram at the study setting is about 250 US dollars). With MDCT being more and more available and accessible in sub-Saharan settings, many cases of PE that could have remained undiagnosed would be depicted, and hence provide more information on the subject.

MDCT evidence for acute PE was more likely to occur in patients with at least one of the following identifiable thromboembolic risk factors: age 65 years and above, known deep vein thrombosis, immobilization due to surgery (mostly caesarean section) or medical reasons and malignancy, with a calculated relative risk of 14.4. Mamlouk et al. had earlier arrived at a similar conclusion [11,12]. On the other hand patients who do not have these risk factors and yet have symptoms indicative of acute PE would be unlikely to have CT pulmonary angiographic findings consistent with acute PE. This might help reduce unnecessary MDCT angiography requests, which would be vital especially in resource-limited settings where patients are often required to pay directly for diagnostic procedures. However, the possibility of depicting any cardiothoracic condition in patients without acute PE or occurring concomitantly with PE may continue to justify routine MDCT angiography in suspected cases of acute PE [14,15]. Symptoms of acute PE may be mild or absent, particularly in patients with PE only in the segmental pulmonary branches, and even in patients with severe PE [11]. Furthermore, symptoms of acute PE are not specific, as other conditions such as lung parenchyma, pleural, cardiac and thoracic vascular abnormalities can equally manifest in the same way. Nevertheless some conditions that mimic acute PE such as occlusion of the coronary arteries remain undiagnosed in the absence of an optimal scanning technique involving ECG-gating. Although the predictive value of normal MDCT is high, normal MDCT angiography does not absolutely rule out pulmonary embolism, thus additional testing is necessary especially when the clinical probability is inconsistent with the imaging results [10].

High radiation exposure with MDCT has been documented [12], and in the absence of other diagnostic techniques in this study setting such as the D-dimer test with a high positive predictive value [12] and perfusion scans, we are left with the patient’s symptoms, thromboembolic risk factors and CT. This emphasizes the need to carefully select patients for MDCT and thus minimize unnecessary patient irradiation [16].

Some aspects that have served as limitations to this study include retrospective data collection and a small sample size. Also no other diagnostic test was performed besides MDCT angiography to further confirm or ascertain the nature of the emboli.

## Conclusion

This study shows that acute PE is a reality in sub-Saharan Africa. The rare occurrence earlier reported can only be associated to the long-term absence of appropriate diagnostic imaging modalities which are sophisticated and costly, requiring trained professionals for optimum results. Patients with clinical suspicion of acute PE with at least a thromboembolic risk factor are more likely to have CT evidence of PE on MDCT pulmonary angiograms. MDCT also retains its unique role in diagnosing concomitant conditions besides PE and will often provide an alternative diagnosis to PE.

## Methods

### Patients

Consecutive records of patients who had MDCT pulmonary angiography performed at the Radiology Department of a university-affiliated hospital (Yaounde General Hospital) from September 2009 to August 2011 were systematically reviewed. Authorization for the study was obtained from the institutional authorities (Yaounde General Hospital Ethics Committee). The MDCT angiograms were requested based on clinical suspicion of acute PE. Wells criteria included leg or calf pain (11%), “an alternative diagnosis is less likely than PE” (24%), tachycardia (19%), recent surgery or immobilization (35%), previous deep vein thrombosis (8%), and malignancy (3%). The most common clinical symptoms were sudden and/or unexplained chest pain, malaise, syncope or shortness of breath.

### MDCT angiography technique

All CT angiograms were performed with a 16-row detector computed tomography (CT) scanner (ECLOS, Hitachi Medical Corporation, Tokyo, Japan). No special patient preparation was required prior to the exam. The patients were positioned head first with both arms raised above the head. Scanning was done in the cephalo-caudal direction in one breath-hold from the thoracic inlet to the upper abdomen with a display field of view (FOV) from rib-to-rib. About 95–97 ml of iodinated contrast material (370 mg I/ml) was administered preferably through an antecubital vein at a rate of 2.5 to 3.5 ml/s using an 18- or 20- gauge cannula and an automated power injector (VISTRON CT™ Injection System). Real-time bolus tracking technique was used with no saline flushes. Scanning parameters were as follows: collimation 0.625 mm ×16; pitch of 1.06; reconstruction index 0.75 mm; peak voltage 120 kV; tube current 175 mA. The images were transferred to a workstation and viewed using the software Hitachi Image Explorer 4.5.1 (Hitachi Medical Corporation). Standard mediastinal and lung windows with real-time ability to modify the window width and level settings were used to optimize vessel visualization. Multiplanar reformation was used when necessary to help differentiate PE from possible artifacts.

The MDCT pulmonary angiographic diagnostic criteria of acute pulmonary embolism consisted of direct visualisation of an endoluminal, low-attenuating non enhancing filling defect in the main pulmonary artery or a primary-, secondary-, or tertiary-order pulmonary artery branch [4]. This filling defect should be complete or partial forming an acute angle with the arterial wall or centrally located [17]. All the CT angiograms were reviewed by two radiologists with over five to thirty-five years of experience. Repeat and technically inadequate MDCT pulmonary angiograms were excluded from the study.

### Data collection and statistical analysis

A questionnaire was used to collect data. Thromboembolic risk factors were assessed from the clinicians’ documentation where available and recorded: age 65 years or older, postoperative state, known deep vein thrombosis DVT), immobilization due to medical reasons and malignancy [10-12]. The presence of PE and other MDCT findings were also noted. Statistical analysis was performed using the software PASW® Statistics 17.0 (SPSS Inc., Chicago, Illinois, USA).

## Abbreviations

FOV: field of view; I: iodine; kV: kilo volts; mA: milli Ampere; MDCT: multi-detector computed tomography; mg: milli gramme; ml: milli litre; mm: milli metre; PE: pulmonary embolism; s: second; SD: standard deviation; SPSS: statistical package for social sciences.

## Competing interests

The authors declare that they have no competing interests.

## Authors’ contributions

JT conceived the study and participated in its design, data collection, statistical analysis and drafting of the manuscript. BM participated in the study design, review of the images, statistical analysis and the drafting of the manuscript. EF participated in the review of the images and proof-reading of the manuscript. EG participated in the review of the images and proof-reading of the manuscript. AGJ participated in the study design and proof-reading of the manuscript. All authors read and approved the final manuscript.

## Pre-publication history

The pre-publication history for this paper can be accessed here:

http://www.biomedcentral.com/1471-2342/12/31/prepub
